# Supplementary material for: Development and evaluation of a novel music-based therapeutic device for upper extremity movement training: A pre-clinical, single-arm trial
Source: PLoS One. 2020 Nov 19;15(11):e0242552. doi: 10.1371/journal.pone.0242552 (PMC7676671; doi:10.1371/journal.pone.0242552)
Supplement: S1 Dataset — (PDF) [file pone.0242552.s001.pdf]

De-Identified Datasets

Development and evaluation of a novel music-based therapeutic device for upper extremity movement training: a pre-clinical, single arm trial

Nina Schaffert[1,3*], Thenille Braun Janzen[2], Roy Ploigt[3], Sebastian Schlüter[3], Veronica Vuong[4], Michael H. Thaut[4]

[1] Department of Movement and Training Science, Institute for Human Movement Science, University of Hamburg, Hamburg, Germany.

[2] Center for Mathematics, Computing and Cognition, Universidade Federal do ABC, São Bernardo do Campo, Brazil.

[3] BeSB GmbH Berlin, Sound Engineering, Berlin, Germany.

[4] Music and Health Science Research Collaboratory, Faculty of Music, University of Toronto, Toronto, Canada.

*Corresponding author
E-mail: nina.schaffert@uni-hamburg.de (NS)

Task 1

ID	TRIAL	CONDITION	SUM_KEY_ERRORS	RT_MEAN	RT_SD
1	1	Random	0	581.75	126.18
1	2	Sequence	0	586.92	106.73
1	3	Random	0	602.83	129.38
1	4	Sequence	0	574.33	95.42
1	5	Sequence	0	536.42	81.87
1	6	Random	0	571.25	90.76
1	7	Random	0	604.33	129.16
1	8	Sequence	0	544.33	80.66
2	1	Random	0	605.75	188.66
2	2	Sequence	0	540.75	75.36
2	3	Random	0	538.75	92.10
2	4	Sequence	2	514.57	69.88
2	5	Sequence	0	495.83	56.59
2	6	Random	0	513.08	59.87
2	7	Random	0	513.00	91.18
2	8	Sequence	0	495.17	58.61
3	1	Random	0	551.58	158.93
3	2	Sequence	0	487.42	67.25
3	3	Random	0	465.00	57.89
3	4	Sequence	0	445.50	57.62
3	5	Sequence	0	456.75	59.38
3	6	Random	0	464.00	44.04
3	7	Random	0	464.58	59.20
3	8	Sequence	0	442.17	59.90
4	1	Random	0	563.92	92.08
4	2	Sequence	0	559.50	100.02
4	3	Random	0	548.92	75.05
4	4	Sequence	0	514.58	79.24
4	5	Sequence	0	512.67	72.09
4	6	Random	0	510.08	52.54
4	7	Random	0	564.92	112.70
4	8	Sequence	0	504.58	73.57
5	1	Random	0	750.92	300.86
5	2	Sequence	0	634.25	172.46
5	3	Random	0	495.00	62.55
5	4	Sequence	0	486.50	64.24
5	5	Sequence	0	508.00	75.21
5	6	Random	0	485.33	94.91
5	7	Random	0	519.00	147.35
5	8	Sequence	0	468.25	68.60
6	1	Random	0	523.25	64.98
6	2	Sequence	0	529.50	66.41
6	3	Random	0	511.42	71.56
6	4	Sequence	0	497.58	68.95
6	5	Sequence	0	507.42	50.82
6	6	Random	0	535.67	85.80

6	7	Random	0	499.42	59.88
6	8	Sequence	0	516.00	72.62
7	1	Random	0	513.33	61.40
7	2	Sequence	0	512.67	74.84
7	3	Random	0	498.58	59.18
7	4	Sequence	0	474.75	61.42
7	5	Sequence	0	492.00	66.06
7	6	Random	0	474.92	60.20
7	7	Random	0	483.58	74.55
7	8	Sequence	0	477.50	54.16
8	1	Random	0	515.58	66.72
8	2	Sequence	0	529.17	77.02
8	3	Random	0	521.08	79.45
8	4	Sequence	0	514.67	71.07
8	5	Sequence	0	501.58	66.79
8	6	Random	0	514.25	53.25
8	7	Random	0	504.25	77.53
8	8	Sequence	0	492.50	71.70
9	1	Random	0	659.67	249.08
9	2	Sequence	0	528.75	79.14
9	3	Random	0	519.25	89.95
9	4	Sequence	0	495.50	74.51
9	5	Sequence	0	483.33	83.81
9	6	Random	0	489.58	64.20
9	7	Random	0	495.83	53.89
9	8	Sequence	0	482.75	59.17
10	1	Random	0	528.92	139.23
10	2	Sequence	0	473.17	72.73
10	3	Random	0	498.50	64.59
10	4	Sequence	0	503.17	104.64
10	5	Sequence	0	468.92	63.33
10	6	Random	0	451.08	47.97
10	7	Random	0	462.50	44.59
10	8	Sequence	0	524.67	191.69
11	1	Random	0	470.83	45.32
11	2	Sequence	0	502.08	89.59
11	3	Random	0	472.17	47.97
11	4	Sequence	0	460.17	51.56
11	5	Sequence	1	466.00	69.33
11	6	Random	0	466.83	116.12
11	7	Random	0	453.08	53.15
11	8	Sequence	0	449.75	33.33
12	1	Random	0	427.17	122.98
12	2	Sequence	0	452.08	138.54
12	3	Random	1	405.85	55.64
12	4	Sequence	0	427.50	91.00
12	5	Sequence	0	429.92	122.71
12	6	Random	0	420.08	84.63

12	7	Random	0	398.83	49.59
12	8	Sequence	0	393.08	62.63
13	1	Random	0	560.00	74.40
13	2	Sequence	0	553.83	63.26
13	3	Random	0	536.58	64.59
13	4	Sequence	0	496.08	78.74
13	5	Sequence	0	520.92	60.30
13	6	Random	0	580.75	159.89
13	7	Random	0	539.58	99.78
13	8	Sequence	0	515.67	77.06
14	1	Random	0	515.50	50.08
14	2	Sequence	0	534.42	104.14
14	3	Random	0	536.08	67.16
14	4	Sequence	0	489.08	54.97
14	5	Sequence	0	500.58	77.88
14	6	Random	0	495.83	71.34
14	7	Random	0	494.58	70.38
14	8	Sequence	0	438.58	82.09
15	1	Random	0	544.17	61.41
15	2	Sequence	0	508.00	66.64
15	3	Random	0	597.25	155.32
15	4	Sequence	0	501.83	45.45
15	5	Sequence	0	496.42	66.21
15	6	Random	0	508.67	117.33
15	7	Random	0	496.42	46.57
15	8	Sequence	0	491.25	67.34
16	1	Random	0	475.50	78.40
16	2	Sequence	0	470.17	77.32
16	3	Random	0	465.08	79.55
16	4	Sequence	0	435.33	64.84
16	5	Sequence	0	531.33	159.09
16	6	Random	0	439.92	65.19
16	7	Random	0	489.67	134.71
16	8	Sequence	0	445.58	67.10
17	1	Random	0	495.33	63.02
17	2	Sequence	0	495.25	51.80
17	3	Random	0	487.75	60.38
17	4	Sequence	0	472.33	61.54
17	5	Sequence	0	488.08	81.15
17	6	Random	0	491.67	111.73
17	7	Random	0	449.42	66.57
17	8	Sequence	0	449.83	44.25
18	1	Random	0	645.58	266.40
18	2	Sequence	1	624.23	167.66
18	3	Random	0	542.00	40.06
18	4	Sequence	0	525.25	61.92
18	5	Sequence	0	508.83	30.85
18	6	Random	0	518.42	44.24

18	7	Random	0	514.67	49.04
18	8	Sequence	0	483.33	45.18
19	1	Random	0	496.83	54.92
19	2	Sequence	0	476.42	75.32
19	3	Random	0	500.08	68.69
19	4	Sequence	0	470.00	60.29
19	5	Sequence	0	484.00	63.71
19	6	Random	0	459.83	63.56
19	7	Random	0	461.17	63.75
19	8	Sequence	0	493.58	53.72
20	1	Random	0	514.08	101.06
20	2	Sequence	0	484.42	110.29
20	3	Random	0	482.42	71.09
20	4	Sequence	0	452.08	59.85
20	5	Sequence	0	474.67	65.23
20	6	Random	0	521.83	49.76
20	7	Random	0	504.33	72.63
20	8	Sequence	0	487.92	61.41
21	1	Random	0	458.42	50.01
21	2	Sequence	0	492.17	62.51
21	3	Random	0	481.67	62.56
21	4	Sequence	0	452.08	59.45
21	5	Sequence	0	463.08	84.70
21	6	Random	0	517.75	152.12
21	7	Random	0	452.50	67.23
21	8	Sequence	0	435.08	51.46

Task 2

ID	SEQ_LENGTH	TEMPO_BPM	SUM_KEY_ERRORS	IRI_MEAN	IRI_SD
1	6	80	0	757.60	8.91
1	7	80	0	742.50	61.96
1	8	80	0	734.57	29.57
1	9	80	1	910.78	462.97
2	6	80	0	728.60	20.55
2	7	80	0	751.17	21.24
2	8	80	0	721.43	49.95
2	9	80	5	782.46	260.03
3	6	80	0	723.80	52.04
3	7	80	0	738.67	44.47
3	8	80	0	715.71	38.92
3	9	80	0	749.00	35.61
4	6	80	0	646.40	59.14
4	7	80	0	646.33	37.29
4	8	80	0	673.57	45.38
4	9	80	3	681.91	190.40
5	6	80	0	683.80	19.43
5	7	80	2	817.13	315.79
5	8	80	0	738.57	39.11
5	9	80	3	759.09	115.95
6	6	80	0	738.80	66.69
6	7	80	0	739.33	59.42
6	8	80	0	725.29	41.19
6	9	80	0	749.13	41.54
7	6	80	0	661.00	25.73
7	7	80	2	654.13	107.60
7	8	80	0	736.29	45.78
7	9	80	0	727.75	34.77
8	6	80	1	685.17	93.05
8	7	80	2	683.25	48.35
8	8	80	0	675.29	41.16
8	9	80	0	709.50	66.68
9	6	80	0	704.60	42.37
9	7	80	1	683.29	60.32
9	8	80	0	717.43	27.60
9	9	80	0	713.13	47.18
10	6	80	0	746.60	65.69
10	7	80	0	733.17	44.94
10	8	80	0	737.86	30.45
10	9	80	0	751.00	37.34
11	6	80	0	733.20	30.31
11	7	80	3	1164.78	1058.47
11	8	80	0	734.86	51.60

11	9	80	0	738.13	140.34
12	6	80	3	937.13	574.56
12	7	80	0	703.33	39.32
12	8	80	0	734.43	41.10
12	9	80	0	739.88	35.43
13	6	80	0	748.40	41.00
13	7	80	0	738.00	50.66
13	8	80	0	727.43	18.27
13	9	80	1	736.89	65.92
14	6	80	1	683.33	163.87
14	7	80	2	658.38	118.05
14	8	80	0	708.29	27.57
14	9	80	3	612.27	177.21
15	6	80	0	738.00	27.29
15	7	80	0	711.00	52.24
15	8	80	0	729.14	40.70
15	9	80	1	739.00	85.30
16	6	80	0	750.40	25.81
16	7	80	0	728.67	78.16
16	8	80	0	707.57	42.15
16	9	80	1	831.11	308.39
17	6	80	0	725.20	19.77
17	7	80	0	733.67	37.72
17	8	80	0	735.43	43.04
17	9	80	0	694.88	21.96
18	6	80	0	742.00	61.28
18	7	80	1	832.14	267.46
18	8	80	0	741.86	34.91
18	9	80	2	774.20	90.48
19	6	80	0	733.00	75.36
19	7	80	0	718.83	29.33
19	8	80	0	711.14	51.78
19	9	80	0	1094.63	1116.69
20	6	80	0	711.20	33.60
20	7	80	0	724.17	67.57
20	8	80	0	722.71	63.47
20	9	80	0	733.50	23.17
21	6	80	0	759.40	20.65
21	7	80	0	739.33	48.78
21	8	80	0	733.57	33.48
21	9	80	0	738.63	26.76
1	6	66	0	895.80	40.95
1	7	66	0	881.67	101.35
1	9	66	1	907.56	65.42
2	6	66	0	886.60	48.41

2	7	66	0	887.83	32.02
2	9	66	0	841.63	52.09
3	6	66	0	888.00	65.78
3	7	66	0	868.17	100.97
3	9	66	0	864.13	42.38
4	6	66	0	823.00	58.17
4	7	66	1	826.71	137.55
4	9	66	0	857.13	85.81
5	6	66	0	897.40	57.33
5	7	66	0	880.50	79.70
5	9	66	0	888.00	31.74
6	6	66	0	917.20	39.69
6	7	66	0	917.00	162.10
6	9	66	0	916.75	70.78
7	6	66	0	865.80	24.78
7	7	66	0	813.67	37.77
7	9	66	0	925.13	66.90
8	6	66	0	884.20	82.17
8	7	66	0	826.00	52.41
8	9	66	0	823.75	82.76
9	6	66	0	859.00	80.25
9	7	66	0	844.00	59.19
9	9	66	1	799.56	69.34
10	6	66	0	883.60	44.60
10	7	66	0	895.00	20.76
10	9	66	0	902.25	34.90
11	6	66	0	896.40	57.06
11	7	66	0	897.67	60.31
11	9	66	1	906.22	44.90
12	6	66	0	902.40	29.61
12	7	66	0	833.50	66.33
12	9	66	0	857.13	34.54
13	6	66	0	898.60	36.81
13	7	66	0	873.83	30.51
13	9	66	0	900.13	35.73
14	6	66	0	863.60	22.96
14	7	66	0	896.83	78.75
14	9	66	3	788.27	242.63
15	6	66	0	890.20	48.99
15	7	66	0	880.33	34.57
15	9	66	1	807.22	143.98
16	6	66	0	873.40	74.45
16	7	66	0	878.00	61.91
16	9	66	0	897.25	27.71
17	6	66	0	886.20	42.10

17	7	66	0	887.50	31.43
17	9	66	0	931.25	59.72
18	6	66	0	897.80	63.97
18	7	66	0	901.00	33.14
18	9	66	0	906.38	61.29
19	6	66	0	878.20	50.99
19	7	66	0	885.17	68.03
19	9	66	1	2067.78	1518.10
20	6	66	0	888.20	62.26
20	7	66	0	885.50	55.45
20	9	66	2	892.50	52.30
21	6	66	0	914.60	11.10
21	7	66	0	910.67	37.24
21	9	66	0	914.13	61.77

Task 3

ID	TRIAL	SUM_KEY_ERRORS	SUM_HAND_ERRORS	IRI_MEAN	IRI_SD
1	1	0	0	741.33	38.00
1	2	0	0	745.00	24.92
1	3	0	0	738.67	49.48
1	4	0	0	724.33	26.31
1	5	0	0	746.00	41.56
1	6	1	0	743.57	39.52
1	7	0	0	733.17	36.64
1	8	1	0	633.29	150.87
2	1	0	0	709.17	21.40
2	2	0	0	842.17	306.18
2	3	1	0	705.14	94.17
2	4	0	0	731.83	32.30
2	5	0	0	717.50	24.79
2	6	0	0	709.50	33.03
2	7	0	0	844.17	514.90
2	8	0	0	730.00	22.41
3	1	0	0	738.00	106.47
3	2	0	0	719.67	22.24
3	3	1	0	708.43	119.10
3	4	0	0	709.17	41.40
3	5	0	0	743.00	50.41
3	6	0	0	718.17	33.67
3	7	0	0	743.83	29.09
3	8	1	0	731.43	41.98
4	1	0	0	628.50	24.83
4	2	0	0	625.33	46.23
4	3	1	0	755.86	85.09
4	4	0	0	759.67	48.47
4	5	10	1	645.25	220.42
4	6	0	0	738.83	15.48
4	7	0	0	684.00	51.73
4	8	0	0	670.33	48.42
5	1	0	0	700.17	58.70
5	2	2	0	1127.25	814.80
5	3	2	0	839.50	247.68
5	4	3	0	1150.56	706.77
5	5	0	0	751.83	32.38
5	6	2	0	741.75	50.12
5	7	1	0	862.00	314.53
5	8	0	0	758.33	64.50
6	1	0	0	729.67	88.36
6	2	0	0	749.83	48.24
6	3	1	0	1052.14	890.61
6	4	2	0	1286.25	1060.86
6	5	0	0	753.50	80.73
6	6	0	0	760.83	59.13

6	7	2	0	1585.13	1843.19
6	8	6	1	2556.58	1493.43
7	1	0	0	745.83	62.19
7	2	0	0	744.83	25.69
7	3	1	0	729.14	78.31
7	4	0	0	750.33	49.88
7	5	0	0	758.17	47.75
7	6	0	0	747.50	38.99
7	7	0	0	739.43	82.77
7	8	0	0	796.33	31.43
8	1	0	0	683.33	60.69
8	2	1	0	667.00	74.56
8	3	3	0	694.44	86.38
8	4	3	0	711.67	185.95
8	5	0	0	693.17	33.69
8	6	0	0	705.83	27.91
8	7	0	0	674.50	45.72
8	8	0	0	679.67	45.88
9	1	2	0	729.25	113.35
9	2	2	0	646.13	160.07
9	3	2	0	737.25	102.34
9	4	1	0	702.14	82.47
9	5	0	1	736.00	30.17
9	6	1	0	702.14	75.98
9	7	2	0	632.63	145.97
9	8	0	0	711.17	23.23
10	1	0	0	740.83	47.80
10	2	0	0	741.17	32.16
10	3	0	0	749.33	41.60
10	4	0	0	750.83	52.51
10	5	0	0	748.33	52.74
10	6	0	0	748.83	34.87
10	7	1	0	733.57	30.69
10	8	0	0	758.33	54.31
11	1	1	0	727.57	91.61
11	2	3	0	735.56	78.53
11	3	1	0	758.00	60.31
11	4	0	0	734.67	47.50
11	5	0	0	766.00	72.45
11	6	2	0	747.38	149.88
11	7	2	0	718.88	53.89
11	8	0	0	721.33	49.28
12	1	1	0	747.57	39.69
12	2	1	0	743.71	47.72
12	3	6	0	742.00	66.64
12	4	0	0	735.50	22.67
12	5	2	0	746.88	32.55
12	6	0	0	739.67	33.72

12	7	2	0	750.00	39.81
12	8	0	0	755.67	93.13
13	1	0	0	735.83	61.15
13	2	0	0	725.83	32.11
13	3	1	0	763.43	178.06
13	4	0	0	705.17	28.22
13	5	1	0	1049.29	840.13
13	6	7	1	1413.08	1205.04
13	7	1	0	747.14	70.22
13	8	6	0	815.17	406.02
14	1	3	0	612.78	262.09
14	2	5	0	602.36	151.71
14	3	2	0	614.00	168.84
14	4	1	0	651.43	155.54
14	5	0	0	743.67	53.68
14	6	2	0	789.88	290.24
14	7	2	0	662.13	97.24
14	8	2	0	669.00	202.91
15	1	0	0	728.50	48.22
15	2	2	0	783.63	247.78
15	3	1	0	658.14	161.78
15	4	0	0	734.00	27.20
15	5	1	0	668.00	119.96
15	6	0	0	724.17	30.75
15	7	1	0	1058.43	848.45
15	8	0	0	721.67	32.84
16	1	0	0	846.50	307.39
16	2	2	0	1133.50	541.07
16	3	3	0	993.89	428.80
16	4	2	0	1019.25	560.28
16	5	0	1	732.14	33.21
16	6	6	0	1353.25	805.57
16	7	2	0	825.13	273.22
16	8	0	0	732.33	34.42
17	1	0	0	753.00	51.99
17	2	4	0	1867.20	2196.61
17	3	1	0	756.14	98.91
17	4	1	0	730.71	110.99
17	5	2	0	752.50	96.12
17	6	1	0	750.86	55.23
17	7	0	0	734.00	38.76
17	8	1	0	739.57	59.44
18	1	1	0	751.14	237.30
18	2	1	0	732.86	113.81
18	3	2	0	902.00	273.18
18	4	1	0	703.14	55.34
18	5	5	0	1076.73	616.70
18	6	0	0	692.00	31.77

18	7	3	0	723.00	239.16
18	8	4	1	535.80	128.16
19	1	0	0	680.33	48.80
19	2	3	0	1012.78	488.66
19	3	1	0	848.86	334.12
19	4	0	0	729.50	35.31
19	5	1	0	735.00	65.17
19	6	2	0	1672.38	680.37
19	7	1	0	735.00	102.71
19	8	0	0	704.33	17.59
20	1	0	0	715.17	32.08
20	2	3	0	743.67	32.20
20	3	3	0	744.56	39.69
20	4	0	0	725.83	15.20
20	5	0	1	726.50	70.66
20	6	2	0	749.00	40.00
20	7	0	0	746.67	39.25
20	8	3	0	826.22	241.58
21	1	0	0	757.50	37.89
21	2	1	0	753.29	41.23
21	3	0	0	740.17	47.80
21	4	0	0	763.33	28.24
21	5	0	0	763.17	34.08
21	6	0	0	749.67	14.67
21	7	0	0	757.83	32.54
21	8	1	0	743.14	33.96

Task 4

ID	FIGURE	FEEDBACK_CONDITION	DIST_Mean	SUM_TIME_DIFF
1	Sinewave	With Feedback	60.78	79015
1	Sinewave	Muted	43.71	65741
1	Sinewave	With Feedback	48.85	67075
1	Sinewave	Muted	31.82	44233
1	Trianglewave	With Feedback	46.52	58668
1	Trianglewave	Muted	59.38	77012
1	Trianglewave	With Feedback	35.37	53193
1	Trianglewave	Muted	51.17	70723
2	Sinewave	Muted	6.14	17571
2	Sinewave	With Feedback	4.81	10431
2	Sinewave	Muted	6.24	12980
2	Sinewave	With Feedback	6.04	11140
2	Trianglewave	With Feedback	5.86	11626
2	Trianglewave	Muted	6.34	12520
2	Trianglewave	With Feedback	11.04	19975
2	Trianglewave	Muted	5.64	12314
3	Sinewave	Muted	4.14	10667
3	Sinewave	With Feedback	3.97	10136
3	Sinewave	Muted	4.13	10968
3	Sinewave	With Feedback	3.91	9518
3	Trianglewave	Muted	5.79	15437
3	Trianglewave	With Feedback	4.35	12391
3	Trianglewave	Muted	4.79	13916
3	Trianglewave	With Feedback	4.31	12802
4	Sinewave	With Feedback	3.63	11499
4	Sinewave	Muted	3.94	11840
4	Sinewave	With Feedback	4.00	9408
4	Sinewave	Muted	4.52	12467
4	Trianglewave	Muted	3.73	12500
4	Trianglewave	With Feedback	552.37	3159018
4	Trianglewave	Muted	4.90	12985
4	Trianglewave	With Feedback	4.12	12039
5	Sinewave	Muted	32.11	77993
5	Sinewave	With Feedback	10.47	22049
5	Sinewave	Muted	7.20	17164
5	Sinewave	With Feedback	12.47	25848
5	Trianglewave	With Feedback	59.63	149960
5	Trianglewave	Muted	22.31	40890
5	Trianglewave	With Feedback	12.52	37657
5	Trianglewave	Muted	21.82	56699
6	Sinewave	Muted	5.53	13211
6	Sinewave	With Feedback	56.66	125662
6	Sinewave	Muted	53.58	108008
6	Sinewave	With Feedback	31.79	75765
6	Trianglewave	Muted	24.37	88654
6	Trianglewave	With Feedback	4.22	12250

6	Trianglewave	Muted	43.46	98398
6	Trianglewave	With Feedback	37.66	83673
7	Sinewave	With Feedback	35.92	45371
7	Sinewave	Muted	25.30	30231
7	Sinewave	With Feedback	83.40	99243
7	Sinewave	Muted	138.08	222315
7	Trianglewave	Muted	7.67	14946
7	Trianglewave	With Feedback	29.84	46961
7	Trianglewave	Muted	28.09	40502
7	Trianglewave	With Feedback	32.91	43444
8	Sinewave	With Feedback	42.06	87451
8	Sinewave	Muted	24.65	59614
8	Sinewave	With Feedback	19.41	43413
8	Sinewave	Muted	26.83	71257
8	Trianglewave	Muted	6.14	15460
8	Trianglewave	With Feedback	4.45	10317
8	Trianglewave	Muted	12.68	27813
8	Trianglewave	With Feedback	14.16	36922
9	Sinewave	With Feedback	4.27	11205
9	Sinewave	Muted	4.65	12258
9	Sinewave	With Feedback	3.67	9523
9	Sinewave	Muted	3.55	8332
9	Trianglewave	With Feedback	4.33	12546
9	Trianglewave	Muted	4.65	13723
9	Trianglewave	With Feedback	5.28	8997
9	Trianglewave	Muted	16.63	38147
10	Sinewave	Muted	3.39	9566
10	Sinewave	With Feedback	3.63	9665
10	Sinewave	Muted	3.38	10722
10	Sinewave	With Feedback	17.96	53895
10	Trianglewave	With Feedback	3.99	12233
10	Trianglewave	Muted	3.24	11513
10	Trianglewave	With Feedback	3.15	11141
10	Trianglewave	Muted	3.45	12297
11	Sinewave	With Feedback	5.19	11452
11	Sinewave	Muted	5.22	11660
11	Sinewave	With Feedback	5.08	9577
11	Sinewave	Muted	29.62	54289
11	Trianglewave	Muted	5.28	13078
11	Trianglewave	With Feedback	7.73	13709
11	Trianglewave	Muted	5.55	11447
11	Trianglewave	With Feedback	5.67	12886
12	Sinewave	With Feedback	467.21	1236699
12	Sinewave	Muted	5.60	8804
12	Sinewave	With Feedback	79.58	133622
12	Sinewave	Muted	3.94	6449
12	Trianglewave	With Feedback	6.38	10368
12	Trianglewave	Muted	5.10	9746

12	Trianglewave	With Feedback	5.02	7962
12	Trianglewave	Muted	6.04	10154
13	Sinewave	Muted	5.65	10089
13	Sinewave	With Feedback	7.53	13392
13	Sinewave	Muted	7.40	15628
13	Sinewave	With Feedback	4.91	9705
13	Trianglewave	With Feedback	7.15	13965
13	Trianglewave	Muted	5.89	11760
13	Trianglewave	With Feedback	5.43	11708
13	Trianglewave	Muted	6.16	11741
14	Sinewave	With Feedback	20.53	26479
14	Sinewave	Muted	26.28	36823
14	Sinewave	With Feedback	29.81	46171
14	Sinewave	Muted	23.84	36552
14	Trianglewave	With Feedback	51.51	77160
14	Trianglewave	Muted	23.93	52182
14	Trianglewave	With Feedback	23.90	33409
14	Trianglewave	Muted	25.51	38498
15	Sinewave	With Feedback	6.18	10794
15	Sinewave	Muted	5.37	9253
15	Sinewave	With Feedback	7.05	9729
15	Sinewave	Muted	5.52	7848
15	Trianglewave	Muted	5.93	11916
15	Trianglewave	With Feedback	5.73	9276
15	Trianglewave	Muted	7.71	12449
15	Trianglewave	With Feedback	6.24	9716
16	Sinewave	Muted	3.95	10132
16	Sinewave	With Feedback	4.45	9808
16	Sinewave	Muted	5.00	10459
16	Sinewave	With Feedback	4.78	9433
16	Trianglewave	Muted	4.74	11678
16	Trianglewave	With Feedback	33.05	69479
16	Trianglewave	Muted	4.44	8996
16	Trianglewave	With Feedback	4.50	9821
17	Sinewave	With Feedback	11.11	32657
17	Sinewave	Muted	28.54	84835
17	Sinewave	With Feedback	15.12	42991
17	Sinewave	Muted	7.99	21313
17	Trianglewave	With Feedback	25.04	78184
17	Trianglewave	Muted	10.62	32039
17	Trianglewave	With Feedback	21.26	65066
17	Trianglewave	Muted	26.31	80579
18	Sinewave	Muted	4.13	11475
18	Sinewave	With Feedback	4.26	11394
18	Sinewave	Muted	3.85	11475
18	Sinewave	With Feedback	5.56	14449
18	Trianglewave	Muted	5.76	16323
18	Trianglewave	With Feedback	6.12	12566

18	Trianglewave	Muted	4.98	15975
18	Trianglewave	With Feedback	4.59	13643
19	Sinewave	Muted	4.41	10554
19	Sinewave	With Feedback	5.42	8164
19	Sinewave	Muted	5.25	10189
19	Sinewave	With Feedback	5.21	8356
19	Trianglewave	With Feedback	4.85	11550
19	Trianglewave	Muted	13.39	26616
19	Trianglewave	With Feedback	19.58	32803
19	Trianglewave	Muted	6.78	12048
20	Sinewave	Muted	24.45	45582
20	Sinewave	With Feedback	23.00	47026
20	Sinewave	Muted	29.20	59284
20	Sinewave	With Feedback	19.66	42445
20	Trianglewave	Muted	87.30	149199
20	Trianglewave	With Feedback	31.79	50413
20	Trianglewave	Muted	23.91	43701
20	Trianglewave	With Feedback	18.71	38930
21	Sinewave	With Feedback	20.41	40352
21	Sinewave	Muted	4.73	10027
21	Sinewave	With Feedback	4.18	7849
21	Sinewave	Muted	4.39	9687
21	Trianglewave	With Feedback	38.72	64461
21	Trianglewave	Muted	5.41	9489
21	Trianglewave	With Feedback	33.91	57334
21	Trianglewave	Muted	4.87	7533
