# Supplementary material for: Development and evaluation of a novel music-based therapeutic device for upper extremity movement training: A pre-clinical, single-arm trial
Source: PLoS One. 2020 Nov 19;15(11):e0242552. doi: 10.1371/journal.pone.0242552 (PMC7676671; doi:10.1371/journal.pone.0242552)
Supplement: S1 File — (PDF) [file pone.0242552.s002.pdf]

Development and evaluation of a novel music-based therapeutic device for upper extremity movement training: a pre-clinical, single arm trial

Nina Schaffert[1,3*], Thenille Braun Janzen[2], Roy Ploigt[3], Sebastian Schlüter[3], Veronica Vuong[4], Michael H. Thaut[4]

[1] Department of Movement and Training Science, Institute for Human Movement Science, University of Hamburg, Hamburg, Germany.

[2] Center for Mathematics, Computing and Cognition, Universidade Federal do ABC, São Bernardo do Campo, Brazil.

[3] BeSB GmbH Berlin, Sound Engineering, Berlin, Germany.

[4] Music and Health Science Research Collaboratory, Faculty of Music, University of Toronto, Toronto, Canada.

*Corresponding author
E-mail: nina.schaffert@uni-hamburg.de (NS)

Description of variable labels and definitions as provided in the SONATA output files.

Task settings:
- **Beats per Minute** (BPM): metronome tempo measured in number of beats per minute.
- **Inter-stimulus interval** (ISI): Time difference between two metronome beats (in ms).
- **Interval**: time window around the metronome beats in which the patient is required to press the keys (in ms).
- **Key**: Number corresponding to the keys of the target sequence (1 to 32, from lower left to upper right).
- **Time**: Time differences between two recorded keypresses (in ms).

Keys mode output:
In the Keys mode, each output line corresponds to one response or keypress recorded from the user's interaction with the device's touchscreen, with the following variables:

- **Metronome Event** (TICK_NO): number of metronome beats presented since the start of the task.
- **Sequence Number** (SEQ_NO): expected order of stimuli in the target sequence. Example: a sequence of 13 stimuli has 13 lines/rows.
- **Sequence Key** (SEQ_KEY): expected key number of the target sequence (1 to 32, from lower left to upper right).
- **Patient Key** (PAT_KEY): patient's actual keypress response (1 to 32, from lower left to upper right).
- **Metronome Time** (TICK_TIME): Time of the metronome presentation (cumulative since the beginning of the task in ms).
- **Patient Time** (PAT_TIME): Time of user's response (cumulative since the beginning of the task in ms).
- **Inter-Response Interval** (IRI): Time interval between successive keypresses (in ms).
- **Synchronization Error** (SE): Time difference between the presentation of the metronome beat and motor response (in ms).

Drawing mode output:
In the Drawing mode, each time a user touches and moves their hand/finger on the touchscreen, a new output line appears with the following variables:

- **Patient X Coordinate** (PAT_X): x-coordinate of the users' input (in pixel, 0 to 1880).
- **Patient Y Coordinate** (PAT_Y): y-coordinate of the users' input (in pixel, 0 to 1040).
- **Target Sequence X Coordinate** (SEQ_X): x-coordinate of the nearest drawing/line target section (-1 if outside the corridor).
- **Target Sequence Y Coordinate** (SEQ_Y): y-coordinate of the nearest drawing/line target section (-1 if outside the corridor).
- **Times Difference** (TIME_DIFF): Time interval between successive coordinate changes (in ms).
- **Movement Distance** (DIST): Distance between the user's input and the nearest drawing/line target section (in pixel).
